# Vaginal Microbiota Composition and HPV Genotype-Specific CIN2+ Risk: A Cross-Sectional Study

**DOI:** 10.3390/diagnostics16091387

**Published:** 2026-05-02

**Authors:** Alexandru Hamod, Razvan Popovici, Mihaela Oancea, Mihaela Grigore, Tudor Lazăr, Ingrid-Andrada Vasilache, Anda Pristavu, Dumitru Gafițanu, Alexandra Cristofor, Adina Tănase, Cristina Mandici, Ana-Maria Grigore, Liliana Strat, Cristian Bucșineanu, Manuela Ciocoiu

**Affiliations:** 1 Grigore T. Popa University of Medicine and Pharmacy, 700115 Iasi, Romania; alexandru.hamod@umfiasi.ro (A.H.); mihaela.grigore@umfiasi.ro (M.G.); cristina-elena.mandici@umfiasi.ro (C.M.); bucsineanu.cristian@yahoo.ro (C.B.); manuela.ciocoiu@umfiasi.ro (M.C.); 2 IInd Department of Obstetrics Gynecology, Iuliu Haţieganu University of Medicine and Pharmacy Cluj-Napoca, 13 Emil Isac, 400023 Cluj-Napoca, Romania; 3 Faculty of Medicine and Biological Sciences, “Ștefan cel Mare” University of Suceava, 720229 Suceava, Romania

**Keywords:** microbiota, CINtec, cervical intraepithelial neoplasia, risk stratification, human papillomavirus

## Abstract

**Background/Objectives**: Emerging evidence links vaginal microbiome dysbiosis with HPV persistence and CIN progression, but whether microbiome markers provide incremental prognostic value beyond molecular triage assays remains unclear. This study aimed to evaluate whether *Lactobacillus* depletion and Shannon diversity improve prediction of biopsy-confirmed CIN2+ and CIN3+ outcomes beyond CINtec and HPV-16 genotyping. **Methods**: This was a secondary analysis of a cross-sectional study including 82 women undergoing cervical screening or follow-up for abnormal cytology. Associations with CIN2+, CIN3+, and CINtec positivity were estimated using modified Poisson regression. Multiplicative interaction between HPV-16 and *Lactobacillus* depletion was formally tested. Incremental discriminative performance was assessed using area under the receiver operating characteristic curve (AUC), DeLong’s test, net reclassification improvement (NRI), and integrated discrimination improvement (IDI). **Results**: HPV-16 was the strongest predictor of CIN2+ (PR = 6.08, *p* < 0.001) and CIN3+ (PR = 5.53, *p* = 0.001). A significant sub-multiplicative interaction indicated that *Lactobacillus* depletion carried its strongest prognostic signal in HPV-16-negative women (CIN3+: PR_interaction = 0.04, *p* = 0.003). Adding microbiome markers to CINtec + HPV-16 significantly improved CIN2+ discrimination (ΔAUC = 0.034, *p* = 0.031), driven by correct downward reclassification of non-events (NRI_non-events = 0.833). When added to HPV-16 + age, IDI for CIN2+ reached 0.092 (*p* = 0.004). **Conclusions**: Vaginal microbiome markers, particularly *Lactobacillus* depletion, provide statistically significant incremental discriminative value for CIN2+ beyond CINtec p16/Ki-67 dual staining and HPV-16 genotyping. Microbiome-based triage may be most impactful in HPV-16-negative women.

## 1. Introduction

Persistent infection with high-risk human papillomavirus (hrHPV) is a known risk factor for developing invasive cervical cancer [1]. HPV infection is extremely common, and one epidemiological model estimated an average lifetime probability of acquiring HPV of 84.6% in women [2]. Although the majority of HPV infections are transient and clear spontaneously through immune responses, a subset persists and can progress through preinvasive high-grade cervical intraepithelial neoplasia (CIN2/3) toward invasive malignancy [3,4]. This aspect highlights the need for modern cervical screening and triage strategies. The global burden remains substantial, with an estimated 604,127 new cervical cancer cases and 341,831 deaths in 2020 [5], and oncogenic HPV16/18 genotypes accounting for approximately 70% of cases [6].

Beyond viral presence, increasing literature data indicates that the cervicovaginal/vaginal microbiota (VMB) may modulate HPV acquisition, persistence, and the development or regression of cervical lesions [7,8]. The female genital tract microbiome is commonly dominated by *Lactobacillus* species that support mucosal homeostasis, and predominant *Lactobacillus* can acidify the cervicovaginal environment, forming a chemical barrier to exogenous microbes and viruses [9,10,11].

On the other hand, vaginal dysbiosis is characterized by reduced *Lactobacillus* and increased community diversity with enrichment of anaerobic taxa and has been associated with higher risk of incident HPV infection, HPV persistence, and high-grade lesions and cancer [7,12,13]. Community state type (CST) frameworks derived from sequencing-based profiling further operationalize these patterns, distinguishing *Lactobacillus*-dominant CSTs from *Lactobacillus*-depleted, anaerobe-enriched CST IV profiles that resemble bacterial vaginosis [11,12,14].

Across observational studies, *Lactobacillus* depletion and higher diversity have been linked to increasing CIN severity and HPV positivity, including overrepresentation of anaerobic taxa such as Gardnerella, Prevotella, Megasphaera, and Sneathia in patients with HPV infection and/or higher-grade disease [15,16,17].

Also, several studies have shown that in patients with untreated CIN2, baseline *Lactobacillus*-dominant microbiomes were more likely to regress, whereas *Lactobacillus* depletion and specific anaerobes (including Megasphaera, Prevotella timonensis, and Gardnerella vaginalis) were associated with CIN2 persistence and slower regression, supporting the hypothesis that VMB composition may function as a prognostic biomarker in selected contexts [18,19].

Because HPV DNA testing is highly sensitive, but with limited specificity when considering the high prevalence and spontaneous clearance of infections, effective triage is essential to identify women at higher immediate risk of clinically relevant precancer while minimizing unnecessary colposcopy and overtreatment [20].

p16/Ki-67 dual-stained cytology (CINtec) was developed to identify abnormal cells based on co-staining of p16 and Ki-67 in the same cell, reflecting HPV-driven cell-cycle deregulation and proliferative activity [21,22]. In one meta-analysis that included 21 studies, dual staining has shown a significantly higher specificity (73% for CIN2+ and 61% for CIN3+) than high-risk HPV testing for detection of high-grade CIN [23].

Large triage studies similarly support clinical utility, including evidence that dual-stained cytology can be more sensitive than Pap cytology for triaging HPV-positive women and that combining dual stain with HPV16/18 genotyping can yield high sensitivity for CIN3+ detection [24,25,26].

Nevertheless, despite extensive work describing associations between VMB profiles and HPV/CIN outcomes, the directionality and causal contribution of microbiome changes remain uncertain, and multiple sources highlight the need for further longitudinal and mechanistic studies to clarify whether disease outcomes are influenced by VMB composition and to define how such measures could be used clinically [27,28].

Also, prior microbiome studies raise an explicit clinical question of whether high-diversity vaginal microbiomes could inform intensified surveillance or be used as a triage tool in women with cervical pathology, indicating a persisting translational gap between microbiome association signals and established molecular triage assays [28,29].

In this context, the aim of the present study is to evaluate whether vaginal microbiota parameters are associated with CINtec positivity, biopsy-confirmed CIN2+ and/or CIN3+ outcomes, and to assess whether these microbiome parameters provide independent predictive information when considered alongside HPV-16 genotyping.

## 2. Materials and Methods

### 2.1. Study Design and Population

This is a secondary analysis of a cross-sectional study [30] including 82 patients undergoing cervical cancer screening or follow-up for abnormal cervical cytology between September 2024 and September 2025. The study was conducted in accordance with the Declaration of Helsinki. The protocol was approved by the local institutional ethics committees (Cuza voda Clinical Hospital of Obstetrics and Gynecology-11630/6 September 2024; Grigore T. Popa University of Medicine and Pharmacy Iasi-480/21 October 2024), and written informed consent was obtained from all participants prior to enrollment.

We included patients with available results for cytology, CINtec^®^ (p16/Ki-67 dual immunostaining), microbiota, HPV genotyping, as well as a corresponding cervical biopsy, who gave their informed consent for participation in the study.

The exclusion criteria comprised absence of a histopathologic diagnosis, incomplete screening test results, prior treatment for cervical intraepithelial neoplasia or cervical cancer, pregnancy, recent antibiotic use or vaginal treatments, or insufficient clinical data.

Participants were recruited consecutively and classified according to vaginal microbiome composition into a *Lactobacillus*-dominant group (n = 62 patients) and a *Lactobacillus*-depleted group (n = 20 patients), defined by a *Lactobacillus* relative abundance threshold of <50% of total bacterial sequences.

### 2.2. Clinical Data Collection

Demographic and clinical data were collected at enrollment, including age, body mass index (BMI), residential setting (urban/rural), smoking history, alcohol consumption, hormonal contraception use, sexually transmitted infection (STI) history, prior HPV infection, immunosuppression status, and HPV vaccination history. Cervical histological diagnosis was determined by colposcopy-directed biopsy and classified as negative/CIN1, CIN2, or CIN3/carcinoma in situ. Two composite binary endpoints were defined: CIN2+ (CIN2 or worse) and CIN3+ (CIN3 or worse).

### 2.3. HPV Genotyping

HPV genotyping was performed on cervical specimens as previously described [30] to identify individual viral genotypes. HPV-16, HPV-18, and other high-risk HPV types were recorded separately (26, 31, 33, 35, 39, 45, 51, 52, 53, 56, 58, 59, 66, 68, 69, 73, 82). Co-infection with multiple HPV strains and the presence of low-risk HPV types were also documented. High-risk HPV positivity was defined as the presence of any oncogenic HPV genotype.

### 2.4. CINtec p16/Ki-67 Dual Staining

CINtec PLUS (CINtec^®^ PLUS Cytology kit, Roche mtm Laboratories AG, Mannheim, Germany) immunocytochemistry was performed on cervical cytology specimens [30]. Results were classified as positive or negative according to manufacturer instructions. CINtec positivity served both as a clinical outcome (in models assessing predictors of epithelial transformation) and as a predictor variable (in models for CIN2+ and CIN3+).

### 2.5. Vaginal Microbiome Profiling

Vaginal samples were collected and subjected to 16S rRNA gene sequencing targeting bacterial genera as previously described [30]. Genus-level taxonomic abundances were obtained for 122 bacterial genera across all 82 participants. Relative abundances were calculated by total sum scaling (TSS), dividing each genus count by the total read count per sample. *Lactobacillus* depletion was defined as a *Lactobacillus* relative abundance below 50% of total bacterial sequences. This threshold was selected based on prior literature characterizing community state types (CSTs) of the vaginal microbiome, where *Lactobacillus*-dominant communities (CST I, II, III, V) typically exhibit relative abundances above 50%, while diverse, anaerobe-rich communities (CST IV) fall below this threshold (11, 12, 14). Also, the 50% relative abundance threshold we applied is directly consistent with the definition used by Tortelli et al. [31], who classified vaginal communities as *Lactobacillus*-dominant when a single *Lactobacillus* species comprised ≥50% of total 16S rRNA sequences.

Vaginal samples were self-collected using the OMNIgene^®^•VAGINAL (OMR-130, DNA Genotek, Ottawa, ON, Canada) nucleic acid stabilization device, which preserves the microbial profile at the time of sampling by preventing post-collection growth and degradation. Microbial DNA was extracted according to the OMNIgene^®^•VAGINAL DNA extraction protocol provided by the manufacturer and quantified prior to library preparation. The full-length 16S rRNA gene was amplified using barcoded primers from the Oxford Nanopore Technologies 16S Barcoding Kit (SQK-16S024)- Oxford Nanopore Technologies, Oxford, United Kingdom, following the ONT 16S Barcoding Kit 1–24 protocol, which includes PCR amplification, AMPure XP bead-based cleanup, and rapid adapter attachment. Libraries were loaded onto R9.4.1 (FLO-MIN106) flow cells and sequenced on the MinION platform according to the ONT MinION sequencing protocol.

Raw nanopore signal data were acquired, base called, and demultiplexed in real time using MinKNOW (Oxford Nanopore Technologies, Oxford, United Kingdom), generating per-sample FASTQ files. Reads were quality-filtered to remove low-quality and short sequences, and taxonomic classification was performed using a 16S rRNA gene reference-based approach, assigning taxa at the genus level. Low-abundance features were filtered to reduce background noise, and normalized relative abundance tables were used for downstream analyses.

### 2.6. Alpha Diversity

Alpha diversity was quantified using the Shannon diversity index, calculated from raw genus-level count data. The Shannon index captures both richness (number of genera present) and evenness (uniformity of their distribution), with higher values indicating more diverse communities.

### 2.7. Beta Diversity and PERMANOVA

Beta diversity was assessed using the Bray–Curtis dissimilarity metric computed on TSS-normalized genus abundances. Compositional differences between clinical groups were tested using permutational multivariate analysis of variance (PERMANOVA) with 999 permutations. Models included CIN2+ status, HPV-16 positivity, and *Lactobacillus* depletion as predictors. HPV-16 was selected as the HPV-related covariate given its dominant role in cervical carcinogenesis and its strongest association with high-grade lesions in the study cohort, while inclusion of broader HPV categories was avoided to reduce collinearity and preserve model interpretability. Homogeneity of multivariate dispersions was evaluated using betadisper to verify the assumption of equal group dispersions.

### 2.8. Differential Abundance Analysis

Differential abundance of individual genera across clinical groups (CIN2+, CIN3+, CINtec positivity) was assessed using centered log-ratio (CLR) transformed abundances. Zero values were replaced using the count zero multiplicative method prior to CLR transformation. Linear models adjusted for age were used to test for associations between genus-level CLR abundances and each outcome, with *p*-values corrected for multiple testing using the Benjamini–Hochberg false discovery rate (FDR) procedure. A significance threshold of q < 0.05 was applied.

### 2.9. Statistical Analysis

Continuous variables were summarized as medians with interquartile ranges (IQRs) and compared between groups using the Wilcoxon rank-sum test. Categorical variables were presented as frequencies and percentages and compared using Fisher’s exact test.

Associations between predictors and binary outcomes (CIN2+, CIN3+, CINtec positivity) were estimated using modified Poisson regression with robust (sandwich) standard errors, yielding prevalence ratios (PR) with 95% confidence intervals. Multiplicative interaction between HPV-16 and *Lactobacillus* depletion was assessed by including a product term (HPV-16 × *Lactobacillus* depletion) in modified Poisson regression models. A significant interaction term with a coefficient below unity was interpreted as a sub-multiplicative (negative) interaction on the multiplicative scale.

The discriminative ability of the evaluated regression models was evaluated using the area under the receiver operating characteristic curve (AUC). Models were built incrementally: (1) CINtec alone, (2) CINtec + HPV-16, (3) CINtec + HPV-16 + *Lactobacillus* depletion, and (4) CINtec + HPV-16 + *Lactobacillus* depletion + Shannon diversity. Pairwise comparison of AUCs was performed using DeLong’s test for correlated ROC curves.

The net reclassification improvement (NRI) and integrated discrimination improvement (IDI) were calculated to quantify how the addition of microbiome markers reclassified individuals compared with base models. Continuous NRI was decomposed into event and non-event components to distinguish improvements in sensitivity from improvements in specificity. IDI was computed with 95% confidence intervals based on asymptotic standard errors.

All statistical analyses were performed in R version 4.5.3 (R Foundation for Statistical Computing, Vienna, Austria). Two-sided *p*-values < 0.05 were considered statistically significant.

## 3. Results

### 3.1. Baseline Characteristics

The study included 82 participants, of whom 62 were classified as having a *Lactobacillus*-dominant vaginal microbiome and 20 as having a *Lactobacillus*-depleted microbiome. The median age of the study population was 40 years (IQR 32–45) (Table 1). Patients with a *Lactobacillus*-depleted microbiome were significantly older than those with a *Lactobacillus*-dominant microbiome (42 vs. 38 years, *p* = 0.020).

Lifestyle-related factors, including smoking history, alcohol consumption, and hormonal contraception use, showed no significant differences between women with *Lactobacillus*-dominant and *Lactobacillus*-depleted microbiomes. Likewise, sexually transmitted infection history, prior HPV infection, and immunosuppression status were similar in both groups.

HPV-16 positivity was significantly more frequent in the *Lactobacillus*-depleted group compared with the *Lactobacillus*-dominant group (55% vs. 26%, *p* = 0.027). Similarly, HPV-18 infection was markedly higher among women with a *Lactobacillus*-depleted microbiome (25% vs. 1.6%, *p* = 0.003). Although the presence of any high-risk HPV type was more common in the *Lactobacillus*-depleted group (95% vs. 76%), this difference did not reach statistical significance (*p* = 0.10). A distribution of other high-risk genotypes according to the microbiome status is presented in Figure S1.

The Shannon diversity index was significantly higher in the *Lactobacillus*-depleted microbiome group compared with the *Lactobacillus*-dominant group (1.31 vs. 0.08, *p* < 0.001), indicating a more diverse bacterial community in the depleted microbiome.

Additionally, CINtec p16/Ki-67 positivity was significantly more frequent among patients with a *Lactobacillus*-depleted microbiome (80% vs. 37%, *p* = 0.002).

### 3.2. Microbiome Characterization in Relationship with the Evaluated Outcomes

**Figure 1:**
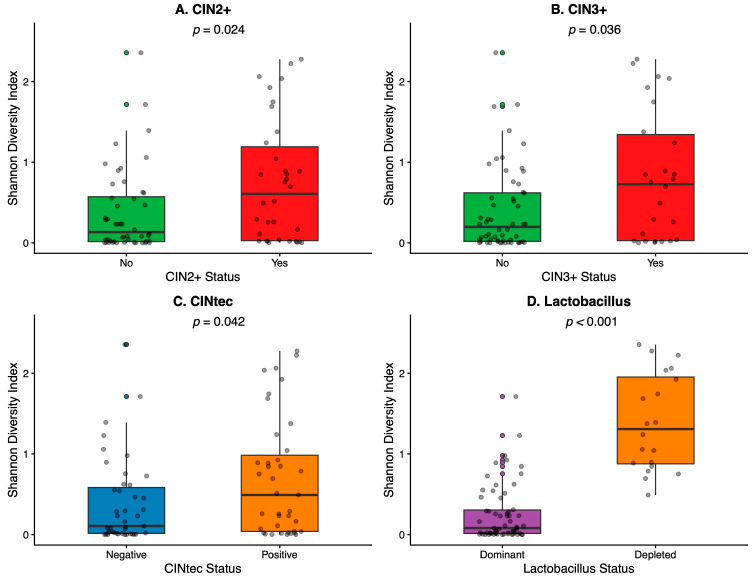
Shannon Diversity by clinical groups.

Shannon diversity was higher in CIN2+ compared with CIN1/negative histology (median 0.605 [IQR 1.164] vs. 0.134 [IQR 0.555], *p* = 0.024) and higher in CIN3+ compared with non-CIN3+ (0.726 [1.316] vs. 0.198 [0.600], *p* = 0.036) (Table 2; Figure 1). Shannon diversity was also higher in CINtec+ compared with CINtec− (0.493 [0.944] vs. 0.107 [0.570], *p* = 0.042) (Table 2; Figure 1). As expected, Shannon diversity was markedly higher in *Lactobacillus*-depleted vs. *Lactobacillus*-dominant microbiomes (*p* = 1.2 × 10^−9^)—Figure 1 and Table 2.

**Figure 2:**
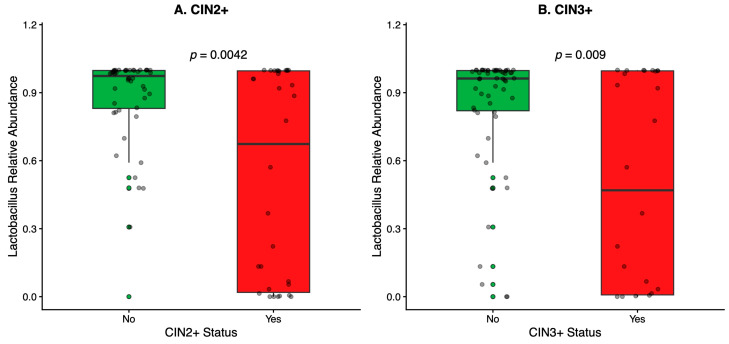
*Lactobacillus* relative abundance by disease status.

*Lactobacillus* relative abundance was lower in CIN2+ compared with CIN1/negative histology (median 0.674 [0.977] vs. 0.974 [0.167], *p* = 0.0042) and lower in CIN3+ compared with non-CIN3+ (0.469 [0.988] vs. 0.963 [0.177], *p* = 0.009) (Table 2; Figure 2).

Exploratory differential abundance analysis showed consistently lower *Lactobacillus* abundance in outcome-positive groups (Table 3). The association was strongest for CIN2+ (coefficient = −2.81, FDR q = 0.008), while similar inverse associations were observed for CIN3+ (coefficient = −2.63, q = 0.061) and CINtec positivity (coefficient = −2.33, q = 0.118). These findings support the broader pattern of *Lactobacillus* depletion in women with higher-risk cervical disease, although only the CIN2+ association remained significant after FDR correction.

**Figure 3:**
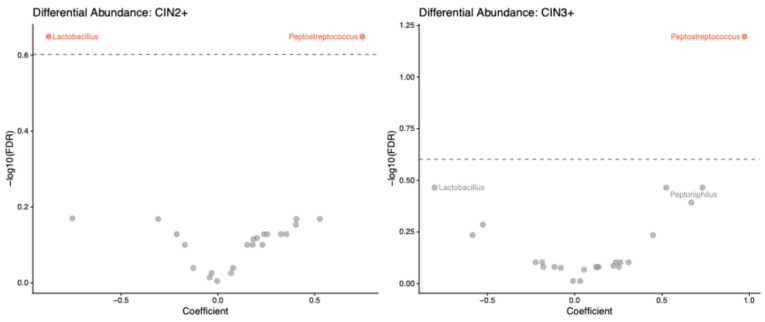
Genus-Level Differential Abundance in CIN2+ and CIN3+ Outcomes.

Differential abundance analysis revealed a consistent dysbiotic pattern characterized by decreased *Lactobacillus* and increased anaerobic taxa, particularly *Peptostreptococcus* (Figure 3). While both taxa were significantly associated with CIN2+, only *Peptostreptococcus* remained significantly associated with CIN3+, suggesting that anaerobic enrichment may be more strongly linked to disease progression than *Lactobacillus* depletion alone.

**Figure 4:**
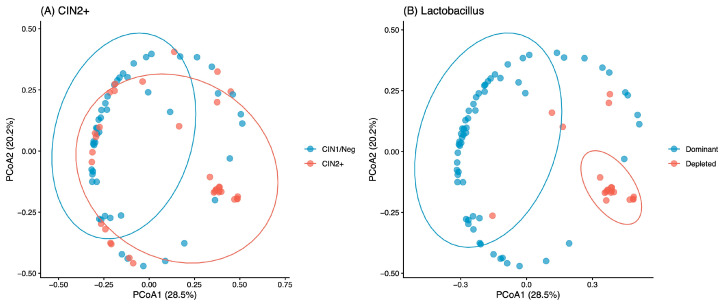
Principal Coordinates Analysis of Vaginal Microbiota by CIN2+ Status and *Lactobacillus* Abundance.

In PERMANOVA (Bray–Curtis, 999 permutations), a model including CIN2+, HPV-16, and *Lactobacillus* depletion explained 18.0% of microbiome compositional variance (R2 = 0.180; F = 5.723; *p* = 0.001) (Figure 4). Homogeneity of group dispersions was evaluated via beta-dispersion analyses (Supplementary Figures S2 and S3).

### 3.3. Multivariable Models for CIN2+ and CIN3+

#### 3.3.1. Modified Poisson Regression (Prevalence Ratios)

In adjusted modified Poisson models including HPV genotype, age, *Lactobacillus* depletion, and Shannon diversity (Table 4), HPV-16 was the strongest independent predictor of both CIN2+ (PR = 6.08, 95% CI: 2.31–16.00, *p* < 0.001) and CIN3+ (PR = 5.53, 95% CI: 2.06–14.84, *p* = 0.001). Neither *Lactobacillus* depletion below 50% (CIN2+: PR = 1.53, 95% CI: 0.81–2.86, *p* = 0.186; CIN3+: PR = 1.34, 95% CI: 0.56–3.20, *p* = 0.516) nor Shannon diversity (CIN2+: PR = 1.28, *p* = 0.350; CIN3+: PR = 1.35, *p* = 0.420) reached statistical significance in the main-effects models. HPV-18 and other high-risk HPV types were also not significantly associated with either outcome.

For CINtec positivity, HPV-16 was strongly associated (PR = 4.15, 95% CI: 2.45–7.04, *p* < 0.001), while other variables such as HPV-18, other HPV-HR, age, *Lactobacillus* depletion and Shannon diversity did not reach statistical significance—Table 5.

#### 3.3.2. Interaction Between HPV-16 and *Lactobacillus* Depletion

A significant negative interaction between HPV-16 and *Lactobacillus* depletion was observed in modified Poisson models, suggesting sub-multiplicative joint effects (Table 6).

In the CIN2+ model, *Lactobacillus* depletion was independently associated with increased risk (PR = 4.72, 95% CI: 1.70–13.07, *p* = 0.003), while the interaction term between HPV-16 and *Lactobacillus* depletion was below unity (PR = 0.28, 95% CI: 0.10–0.80, *p* = 0.017). A similar but more pronounced pattern was observed for CIN3+, where *Lactobacillus* depletion alone was associated with a markedly increased risk (PR = 20.31, 95% CI: 2.66–155.11, *p* = 0.004), and the interaction term was PR = 0.04 (95% CI: 0.005–0.36, *p* = 0.004).

### 3.4. Incremental Discriminative Value of Microbiome Markers

To assess whether vaginal microbiome markers add clinically meaningful discrimination beyond established biomarkers, we compared regression models (Table 7). CINtec alone achieved an AUC of 0.898 for CIN2+ and 0.884 for CIN3+. Adding HPV-16 improved discrimination modestly (CIN2+: AUC = 0.919; CIN3+: AUC = 0.911).

**Figure 5:**
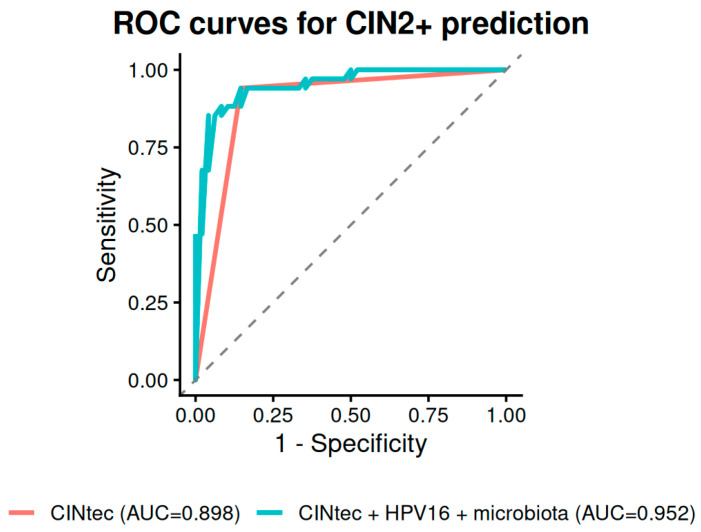
ROC Curve Comparison for CIN2+ Prediction Models.

**Figure 6:**
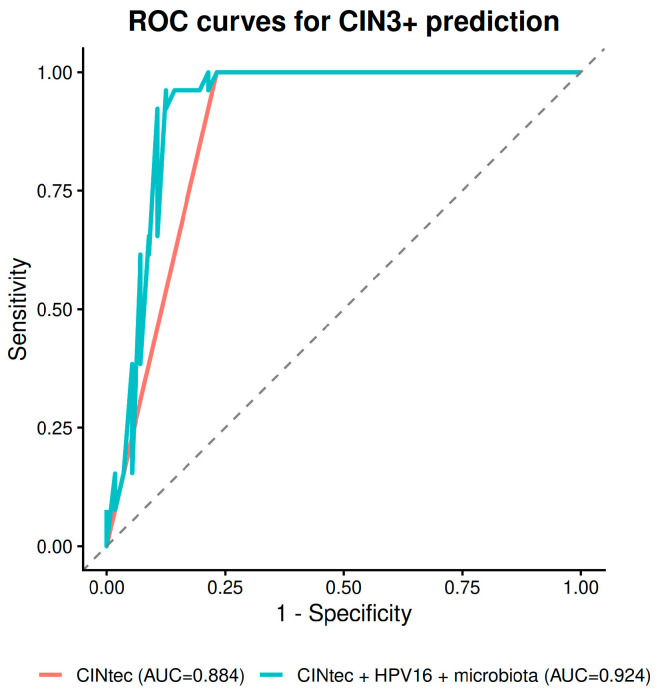
ROC Curve Comparison for CIN3+ Prediction Models.

The addition of *Lactobacillus* depletion status further increased the CIN2+ AUC to 0.941, and including Shannon diversity yielded a final AUC of 0.952 (Figure 5). The model which comprised CINtec, HPV-16 and microbiome parameters achieved the best discriminative power for CIN3+ (AUC = 0.931/0.924)—Figure 6. The improvement from the CINtec + HPV-16 model to the full model including microbiome markers was statistically significant for CIN2+ (ΔAUC = 0.034, DeLong *p* = 0.031), but not for CIN3+ (ΔAUC = 0.014, *p* = 0.453).

The reclassification performance of microbiome-enhanced models was assessed using continuous Net Reclassification Improvement (NRI) and Integrated Discrimination Improvement (IDI) (Table 8).

When *Lactobacillus* depletion and Shannon diversity were added to a base model containing CINtec and HPV-16, the total NRI was 0.775 for CIN2+ and 0.571 for CIN3+. This reclassification was driven almost entirely by correct downward reclassification of non-events (NRI_non-events = 0.833 and 0.571, respectively), indicating that microbiome markers primarily improve identification of patients at low risk. On the other hand, reclassification of events was minimal (CIN2+: NRI_events = −0.059; CIN3+: NRI_events = 0.000), suggesting no improvement in risk classification of true cases beyond the CINtec + HPV-16 model. IDI values were modest and did not reach statistical significance (CIN2+: IDI = 0.039, *p* = 0.077; CIN3+: IDI = 0.046, *p* = 0.080).

When microbiome markers were added to a simpler base model containing only HPV-16 and age, reclassification gains were more pronounced. For CIN2+, IDI reached 0.092 (95% CI: 0.026–0.163, *p* = 0.004), indicating a statistically significant improvement in discrimination. The total NRI for CIN2+ was again 0.775, driven by improved classification of non-events (NRI_non-events = 0.833). For CIN3+, both event (NRI_events = 0.154) and non-event (NRI_non-events = 0.536) classification improved, with a total NRI of 0.690, although IDI did not reach statistical significance (IDI = 0.052, *p* = 0.087).

## 4. Discussion

This study investigated whether vaginal microbiome markers such as *Lactobacillus* depletion and Shannon diversity add independent prognostic value for high-grade cervical intraepithelial neoplasia (CIN2+, CIN3+) beyond established biomarkers including HPV-16 genotyping and CINtec p16/Ki-67 dual staining.

Three principal findings emerged from our secondary analysis. First, HPV-16 was the dominant predictor of both CIN2+ and CIN3+ across all modeling approaches, consistent with its established role as the most oncogenic HPV genotype. Multiple studies confirmed that high-risk genotypes, especially HPV16, are the strongest viral predictors of CIN2+ and CIN3+ [16,32,33].

Second, a significant sub-multiplicative interaction between HPV-16 and *Lactobacillus* depletion indicated that vaginal dysbiosis carries its strongest prognostic signal in HPV-16-negative women. While most microbiome papers do not formally model statistical interaction with HPV16, several studies reported that vaginal dysbiosis adds risk information particularly where viral markers alone are less informative [16]. In the Costa Rica Vaccine Trial, higher Shannon diversity at a later visit independently predicted CIN2+ (OR 1.19 per unit), even after accounting for hrHPV infection status [34]. Also, baseline Gardnerella promoted progression via increased subsequent diversity. Moreover, a cross-sectional study found that bacterial vaginosis and hrHPV were independent risk factors for CIN in multivariable models, suggesting the vaginal milieu modifies risk on top of viral genotype [35].

Third, the addition of microbiome markers to models already containing CINtec and HPV-16 significantly improved discriminative performance for CIN2+ (ΔAUC = 0.034, *p* = 0.031), primarily by improving risk stratification among non-events. Several studies indicated that microbiome parameters could offer incremental prognostic value beyond standard molecular triage. A machine-learning model using vaginal microbiome features differentiated CIN2+ vs. CIN1− with AUC 0.95, indicating that bacterial signatures substantially enhance classification [36]. Also, a meta-analysis identified a genus-level signature that distinguished cervical cancer from controls with AUC 0.89, suggesting that microbiome profiles meaningfully improve discrimination [17].

Our finding that *Lactobacillus* depletion and elevated Shannon diversity are associated with more severe cervical disease is consistent with a substantial body of prior literature. In a meta-analysis of 11 studies comprising 1230 cases, Wang et al. reported that cervicovaginal *Lactobacillus* spp. were associated with decreased detection of high-risk HPV (OR = 0.64, 95% CI: 0.48–0.87), CIN (OR = 0.53, 95% CI: 0.34–0.83), and cervical cancer (OR = 0.12, 95% CI: 0.04–0.36) [37]. A larger meta-analysis by Brusselaers et al., pooling 15 prospective studies with over 100,000 women, confirmed that vaginal dysbiosis increased the risk of high-grade lesions and cancer (relative risk, RR = 2.01, 95% CI: 1.40–3.01) [7]. Mitra et al. demonstrated in a cohort of 169 women that the prevalence of high-diversity, low-*Lactobacillus* communities (CST IV) increased progressively with CIN severity, from 10% in healthy controls to 21% in LSIL, 27% in HSIL, and 40% in invasive cervical cancer, and that the vaginal microbiome in HSIL was characterized by higher levels of *Sneathia sanguinegens* and *Peptostreptococcus anaerobius* [38].

Our observation that *Peptostreptococcus* was the most consistently differentially abundant genus across both CIN2+ and CIN3+ outcomes aligns with these findings and with multiple systematic reviews identifying this taxon among the anaerobes enriched in HPV-associated cervical disease [8,39,40].

The significant Shannon diversity gradient we observed across clinical outcomes (CIN2+ > CIN1/negative; CIN3+ > non-CIN3+; CINtec+ > CINtec−) is a hallmark of the transition from *Lactobacillus*-dominated communities to the polymicrobial, anaerobe-rich CST IV state described across multiple populations. Longitudinal data from Brotman et al. demonstrated that *Lactobacillus*-dominated communities (particularly *L. gasseri*) were associated with faster HPV clearance, while low-*Lactobacillus* CST IV-B communities showed the slowest clearance rates [41]. These temporal dynamics provide a mechanistic framework for interpreting our cross-sectional associations. Thus, *Lactobacillus* depletion may facilitate HPV persistence and thereby promote the sustained oncogene expression necessary for CIN progression.

A novel aspect of our study is the demonstration of a statistically significant sub-multiplicative interaction between HPV-16 and *Lactobacillus* depletion. The interaction was most pronounced for CIN3+, where the prevalence ratio for *Lactobacillus* depletion alone reached 22.62 (*p* = 0.002), while the interaction term was 0.04 (*p* = 0.003). This pattern suggests that in the absence of HPV-16, vaginal dysbiosis may serve as a particularly strong risk factor, potentially operating through alternative pathways involving chronic inflammation, epithelial barrier disruption, or promotion of other high-risk HPV types [42]. Conversely, when HPV-16 is present, its potent oncogenic properties may dominate the carcinogenic process, reducing the relative contribution of the microbial milieu. Liu et al. provided supporting evidence for this framework, demonstrating that the relationship between vaginal microbiota composition and HPV oncogene expression varies by disease stage, with the strongest microbial correlations observed in earlier lesions rather than in established cancers [43].

To our knowledge, this is one of the first studies to formally quantify the incremental discriminative and reclassification value of vaginal microbiome markers beyond CINtec p16/Ki-67 dual staining. CINtec has emerged as a robust triage tool, with literature data showing sensitivity of 87.6% for CIN2+ in HPV-positive women, and specificity approaching 99% in some cohorts.

In our data, CINtec alone achieved AUCs of 0.898 for CIN2+ and 0.884 for CIN3+, confirming its strong baseline performance. Adding HPV-16 genotyping and microbiome markers further improved the CIN2+ AUC to 0.952.

The reclassification analysis provided additional granularity: the total NRI of 0.775 for CIN2+ was driven almost entirely by correct downward reclassification of non-events (NRInon-events = 0.833), indicating that microbiome markers primarily improve identification of low-risk women who could safely avoid colposcopy. This “rule-out” profile is clinically significant in the context of cervical screening, where over-referral to colposcopy represents a major burden in terms of healthcare costs, patient anxiety, and potential for overtreatment.

The clinical implication of these findings is that vaginal microbiome profiling could serve as an adjunct to existing triage algorithms, particularly in settings where CINtec is unavailable. When microbiome markers were added to a simpler HPV-16 + age model, the IDI for CIN2+ was 0.092 (*p* = 0.004), a statistically significant improvement. As 16S rRNA sequencing becomes more accessible and affordable, incorporating a simple binary classifier based on *Lactobacillus* relative abundance could complement molecular HPV testing without requiring additional clinical procedures.

Several limitations should be acknowledged. First, the cross-sectional design precludes establishing temporal or causal relationships between vaginal dysbiosis and CIN progression. Longitudinal studies with serial microbiome sampling before and after CIN diagnosis are needed to determine whether *Lactobacillus* depletion precedes or follows lesion development.

Second, the sample size of 82 participants, with 20 in the *Lactobacillus*-depleted group, limited statistical power, particularly for interaction analyses.

Third, genus-level 16S rRNA sequencing could not distinguish between *Lactobacillus* species, an important distinction given that *L. crispatus* and *L. iners* exert divergent effects on HPV persistence and cervical disease.

Fourth, the single-center design and the recruitment of women already undergoing screening or follow-up for abnormal cytology limit generalizability to population-based screening cohorts.

## 5. Conclusions

In conclusion, this study demonstrates that vaginal microbiome markers, particularly *Lactobacillus* depletion, provide incremental prognostic value for CIN2+ beyond CINtec p16/Ki-67 dual staining and HPV-16 genotyping.

The significant sub-multiplicative interaction between HPV-16 and vaginal dysbiosis suggests that microbiome-based risk stratification may be most impactful in HPV-16-negative women, a subgroup where conventional triage markers have lower sensitivity.

Future multi-center, longitudinal studies incorporating species-level microbiome profiling are warranted to validate these findings and determine whether microbiome-guided triage can reduce unnecessary colposcopy referrals without compromising sensitivity for high-grade cervical disease.

## Figures and Tables

**Table 1:** Baseline characteristics of the study population according to microbiome status.

Characteristic	Overall (n = 82)	Dominant (n = 62)	Depleted (n = 20)	*p*-Value
Age (years)	40 (32, 45)	38 (29, 43)	42 (39, 51)	0.020
BMI (kg/m^2^)	24.4 (21.8, 28.0)	23.8 (21.7, 27.6)	25.2 (22.6, 29.5)	0.3
Urban	64 (78%)	51 (82%)	13 (65%)	0.13
Rural	18 (22%)	11 (18%)	7 (35%)	
Smoking history	39 (48%)	29 (47%)	10 (50%)	>0.9
Alcohol consumption	7 (8.5%)	6 (9.7%)	1 (5.0%)	>0.9
Hormonal contraception	21 (26%)	16 (26%)	5 (25%)	>0.9
HPV vaccination	20 (24%)	18 (29%)	2 (10%)	0.13
STI history	3 (3.7%)	2 (3.2%)	1 (5.0%)	>0.9
Previous HPV history	65 (79%)	48 (77%)	17 (85%)	0.5
Immunosuppression	2 (2.4%)	2 (3.2%)	0 (0%)	>0.9
HPV-16 positive	27 (33%)	16 (26%)	11 (55%)	0.027
HPV-18 positive	6 (7.3%)	1 (1.6%)	5 (25%)	0.003
Multiple HPV strains	30 (37%)	21 (34%)	9 (45%)	0.4
Any high-risk HPV	66 (80%)	47 (76%)	19 (95%)	0.10
Low-risk HPV present	6 (7.3%)	5 (8.1%)	1 (5.0%)	>0.9
Shannon diversity index	0.25 (0.02, 0.85)	0.08 (0.01, 0.31)	1.31 (0.87, 1.98)	<0.001
CINtec p16/Ki-67 positive	39 (48%)	23 (37%)	16 (80%)	0.002

Legend: BMI—body mass index; HPV—human papillomavirus; HPV-16/HPV-18—specific HPV genotypes; STI—sexually transmitted infection; *p*-value—statistical significance value; CINtec p16/Ki-67—immunocytochemical test for p16 and Ki-67 protein expression. Note: Values are presented as median (Q1, Q3) or n (%). Wilcoxon rank-sum test was used for continuous variables and Fisher’s exact test for categorical variables.

**Table 2:** Alpha diversity and *Lactobacillus* relative abundance across clinical groups (Wilcoxon rank-sum tests).

Comparison	Metric	Group 1 Median (IQR)	Group 2 Median (IQR)	*p*-Value
CIN2+ vs. CIN1/Negative	Shannon diversity index	0.605 (1.164)	0.134 (0.555)	0.024
CIN3+ vs. non-CIN3+	Shannon diversity index	0.726 (1.316)	0.198 (0.600)	0.036
CINtec+ vs. CINtec−	Shannon diversity index	0.493 (0.944)	0.107 (0.570)	0.042
*Lactobacillus*-depleted vs. *Lactobacillus*-dominant	Shannon diversity index	—	—	1.2 × 10^−9^
CIN2+ vs. CIN1/Negative	*Lactobacillus* relative abundance	0.674 (0.977)	0.974 (0.167)	0.004
CIN3+ vs. non-CIN3+	*Lactobacillus* relative abundance	0.469 (0.988)	0.963 (0.177)	0.009

Legend: IQR—interquartile range; CIN—cervical intraepithelial neoplasia; CIN2+/CIN3+—cervical intraepithelial neoplasia grade 2 or higher/grade 3 or higher; CIN1—cervical intraepithelial neoplasia grade 1; CINtec—dual-stain test for p16/Ki-67.

**Table 3:** Association between *Lactobacillus* abundance and high-grade cervical lesion outcomes.

Outcome	Genus	Coefficient	Raw *p*-Value	FDR q-Value
CIN2+	*Lactobacillus*	−2.81	<0.001	0.008
CIN3+	*Lactobacillus*	−2.63	<0.001	0.061
CINtec positivity	*Lactobacillus*	−2.33	<0.001	0.118

Legend: CIN—cervical intraepithelial neoplasia; CIN2+/CIN3+—cervical intraepithelial neoplasia grade 2 or higher/grade 3 or higher; CINtec—dual-stain test for p16/Ki-67; FDR—false discovery rate; q-value—FDR-adjusted *p*-value.

**Table 4:** Modified Poisson regression: CIN2+ and CIN3+.

Predictor	CIN2+ PR (95% CI)	*p*	CIN3+ PR (95% CI)	*p*
HPV-16	6.08 (2.31 –16.00)	<0.001	5.53 (2.06–14.84)	0.001
HPV-18	1.19 (0.70 –2.01)	0.519	1.44 (0.77–2.69)	0.257
Other HR-HPV	2.04 (0.71 –5.84)	0.184	0.99 (0.26–3.73)	0.984
Age	1.00 (0.98 –1.02)	0.866	1.00 (0.97 –1.03)	0.851
*Lactobacillus* depletion (<50%)	1.53 (0.81 –2.86)	0.186	1.34 (0.56 –3.20)	0.516
Shannon diversity	1.28 (0.76 –2.15)	0.350	1.35 (0.65 –2.82)	0.420

Legend: PR—prevalence ratio; CI—confidence interval; CIN—cervical intraepithelial neoplasia; CIN2+/CIN3+—cervical intraepithelial neoplasia grade 2 or higher/grade 3 or higher; HPV—human papillomavirus; HPV-16/HPV-18—specific high-risk HPV genotypes; HR-HPV—high-risk human papillomavirus (other than 16/18 types).

**Table 5:** Modified Poisson regression: CINtec positivity.

Predictor	PR (95% CI)	*p*-Value
HPV-16	4.15 (2.45–7.04)	<0.001
HPV-18	1.04 (0.63–1.73)	0.867
Other HR-HPV	1.29 (0.88–1.90)	0.185
Age	0.98 (0.96–1.01)	0.146
*Lactobacillus* depletion (<50%)	1.23 (0.71–2.12)	0.459
Shannon diversity	1.37 (0.91–2.06)	0.133

Legend: PR—prevalence ratio; CI—confidence interval; HPV—human papillomavirus; HPV-16—high-risk human papillomavirus genotype 16.

**Table 6:** Modified Poisson regression with interaction models: HPV-16 × *Lactobacillus* depletion.

Predictor	CIN2+ PR (95% CI)	*p*	CIN3+ PR (95% CI)	*p*
HPV-16	6.43 (2.67–15.46)	<0.001	31.96 (4.17–245.18)	<0.001
Age	1.00 (0.98–1.03)	0.770	1.02 (1.00–1.05)	0.116
*Lactobacillus* depletion	4.72 (1.70–13.07)	0.003	20.31 (2.66–155.11)	0.004
HPV-16 × *Lactobacillus* depletion	0.28 (0.10–0.82)	0.020	0.04 (0.005–0.36)	0.004
HPV-18	1.06 (0.71–1.58)	0.784	1.29 (0.82–2.03)	0.275

Legend: PR—prevalence ratio; CI—confidence interval; CIN—cervical intraepithelial neoplasia; CIN2+/CIN3+—cervical intraepithelial neoplasia grade 2 or higher/grade 3 or higher; HPV—human papillomavirus; HPV-16—high-risk human papillomavirus genotype 16.

**Table 7:** Incremental discriminative performance.

Model	CIN2+ AUC	CIN3+ AUC
CINtec	0.898	0.884
CINtec + HPV-16	0.919	0.911
CINtec + HPV-16 + *Lactobacillus* depletion	0.941	0.931
CINtec + HPV-16 + *Lactobacillus* depletion + Shannon diversity	0.952	0.924
DeLong *p*	0.031	0.453

Legend: AUC—area under the receiver operating characteristic curve; CIN—cervical intraepithelial neoplasia; CIN2+/CIN3+—cervical intraepithelial neoplasia grade 2 or higher/grade 3 or higher; CINtec—dual-stain test for p16/Ki-67; HPV—human papillomavirus; HPV-16—high-risk human papillomavirus genotype 16.

**Table 8:** Net Reclassification Improvement (NRI) and Integrated Discrimination Improvement (IDI).

Model	Endpoint	NRI (Events)	NRI (Non-Events)	NRI Total	IDI	95% CI (IDI)	*p*-Value
CINtec + HPV16 vs. + microbiota	CIN2+	−0.059	0.833	0.775	0.039	−0.004 to 0.081	0.077
CINtec + HPV16 vs. + microbiota	CIN3+	0.000	0.571	0.571	0.046	−0.008 to 0.101	0.080
HPV16 + age vs. + microbiota	CIN2+	−0.059	0.833	0.775	0.092	0.026 to 0.163	0.004
HPV16 + age vs. + microbiota	CIN3+	0.154	0.536	0.690	0.052	−0.007 to 0.114	0.087

Legend: NRI—net reclassification improvement; IDI—integrated discrimination improvement; CI—confidence interval; CIN—cervical intraepithelial neoplasia; CIN2+/CIN3+—cervical intraepithelial neoplasia grade 2 or higher/grade 3 or higher; HPV—human papillomavirus; HPV16—high-risk human papillomavirus genotype 16.

## Data Availability

The data presented in this study are available on request from the corresponding author. The data are not publicly available due to local regulations.

## Supplementary Materials

The following supporting information can be downloaded at: https://www.mdpi.com/article/10.3390/diagnostics16091387/s1, Figure S1: Distribution of other high-risk HPV genotypes; Figure S2: Beta-dispersion analysis of vaginal microbiome composition comparing CIN2+ and CIN1/negative histology groups; Figure S3: Beta-dispersion analysis comparing vaginal microbiome variability in CIN3+ versus non-CIN3+ patients.

## Abbreviations

The following abbreviations are used in this manuscript:

AUC	Area under the receiver operating characteristic curve
BMI	Body mass index
CI	Confidence interval
CIN	Cervical intraepithelial neoplasia
CIN2+	Cervical intraepithelial neoplasia grade 2 or higher
CIN3+	Cervical intraepithelial neoplasia grade 3 or higher
CINtec	p16/Ki-67 dual-stain immunocytochemistry test (CINtec PLUS)
CLR	Centered log-ratio
CST	Community state type
FDR	False discovery rate
HPV	Human papillomavirus
hrHPV	High-risk human papillomavirus
HSIL	High-grade squamous intraepithelial lesion
IDI	Integrated discrimination improvement
IQR	Interquartile range
LSIL	Low-grade squamous intraepithelial lesion
NRI	Net reclassification improvement
OR	Odds ratio
PERMANOVA	Permutational multivariate analysis of variance
PR	Prevalence ratio
ROC	Receiver operating characteristic
RR	Relative risk/risk ratio
STI	Sexually transmitted infection
TSS	Total sum scaling
VMB	Vaginal microbiota
