# Supplementary figures and images for: Vaginal Microbiota Composition and HPV Genotype-Specific CIN2+ Risk: A Cross-Sectional Study
Source: Diagnostics (Basel). 2026 May 2;16(9):1387. doi: 10.3390/diagnostics16091387 (PMC13163743; doi:10.3390/diagnostics16091387)

# Betadisper: CIN3+

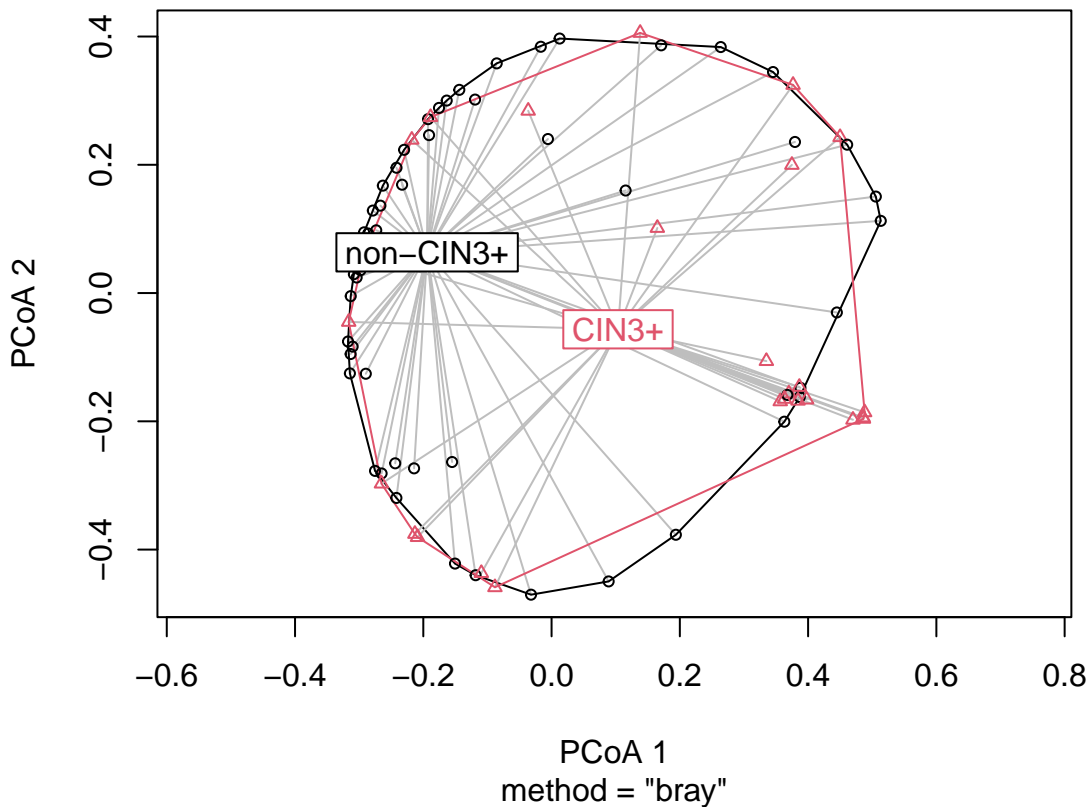

Supplement: Supplementary file 1 [file diagnostics-16-01387-s001.zip › Supplementary figures /Supplement_Betadisper_CIN3.pdf]

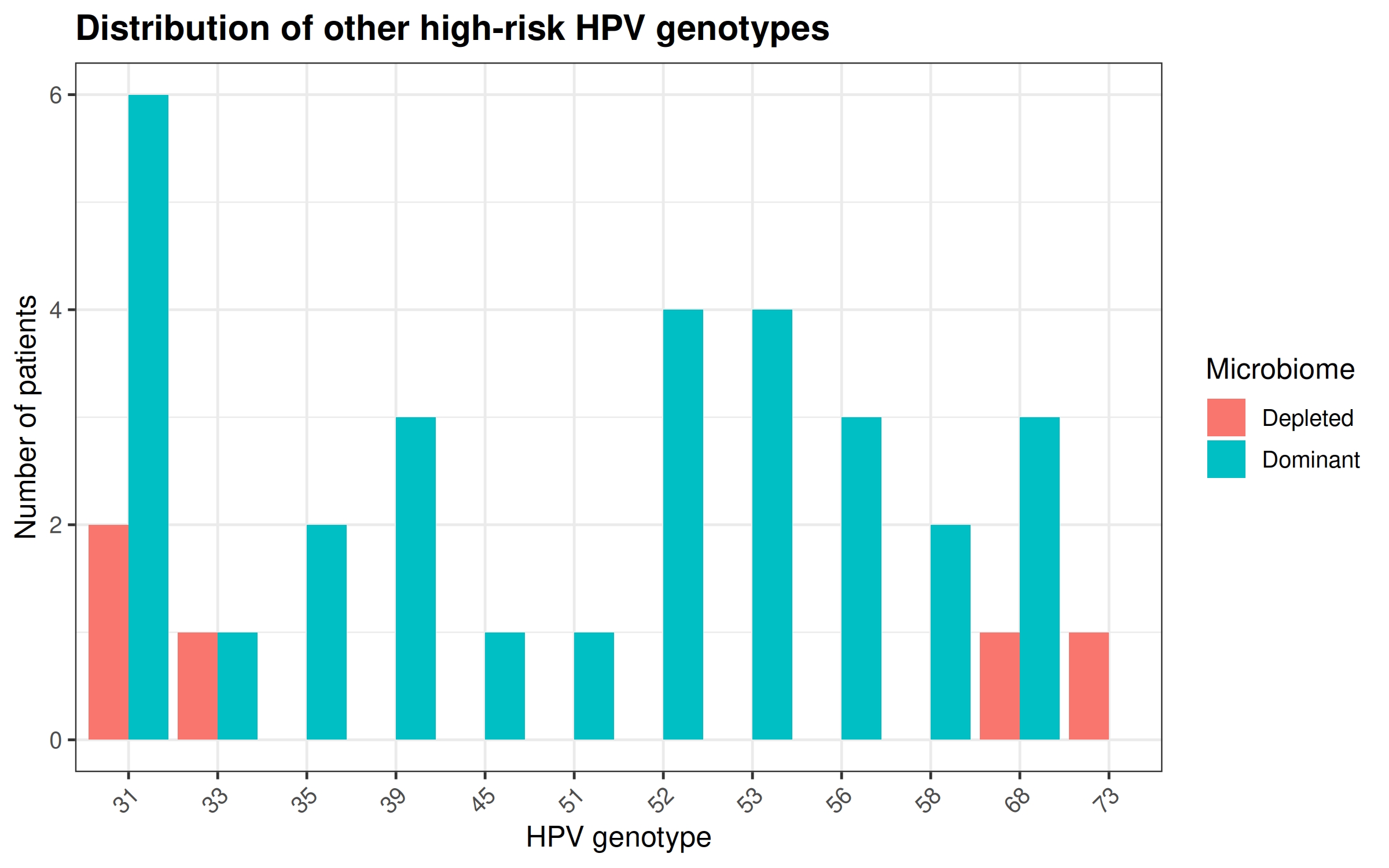

Supplement: Supplementary file 1 [file diagnostics-16-01387-s001.zip › Supplementary figures /Supplementary S1.jpg]

## Betadisper: CIN2+

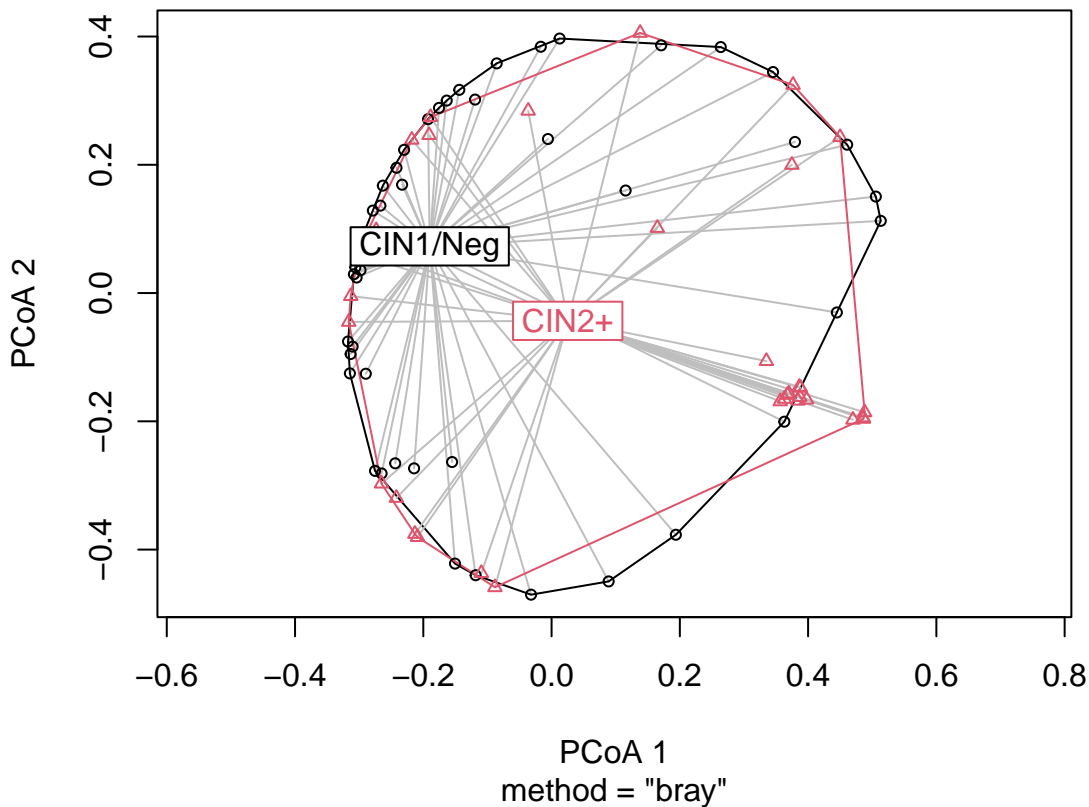

Supplement: Supplementary file 1 [file diagnostics-16-01387-s001.zip › Supplementary figures /Supplement_Betadisper_CIN2.pdf]
